# Optimal Gestational Weight Gain for Tibetans Based on Prepregnancy Body Mass Index

**DOI:** 10.1038/s41598-020-65725-3

**Published:** 2020-07-01

**Authors:** Dajie Chen, Xianxian Zhou, Shijiao Yan, Wenzhen Li, Xueyi Yang, Chuanzhu Lv, Zuxun Lu

**Affiliations:** 1 Department of Social Medicine and Health Management, School of Public Health, Tongji Medical College, Huazhong University of Science and Technology, Wuhan, Hubei 430030 China; 2 Department of Obstetrics and Gynecology, Lhasa General Hospital, Lhasa, Tibet 850000 China; 3 School of International Education, Hainan Medical University, Haikou, Hainan, 571199 China; 4 Emergency and Trauma College, Hainan Medical University, Haikou, Hainan 571199 China

**Keywords:** Epidemiology, Weight management

## Abstract

We aimed to estimate the optimal gestational weight gain (GWG) in Tibetan women and to evaluate the appropriateness of the Institute of Medicine (IOM) GWG recommendations for Tibetan women. We analyzed data from 1474 Tibetan women from a cross-sectional study conducted in 2019. According to the three different body mass index (BMI) classification criteria (WHO BMI categories, WHO Asian BMI categories, Chinese BMI categories), we estimated the association of GWG with pregnancy outcomes (neonate birthweight and mode of delivery) using a polynomial regression model, and the optimal GWG in each BMI group was calculated. The risk of adverse outcomes showed a U-shaped curve with increasing GWG. The optimal GWG of Tibetan women based on the WHO BMI categories was 17.2 kg (range, 13.3 to 20.9 kg) for underweight, 9.3 kg (5.8 to 12.9 kg) for normal weight, and 5.2 kg (1.3 to 9.1 kg) for overweight. Underweight Tibetan mothers may gain more gestational weight than recommend by the IOM guidelines. However, normal weight Tibetan mothers are likely to benefit from gaining less weight than that recommended by the IOM. The GWG recommendations based on the IOM guidelines might not be appropriate for Tibetan women, and ethnicity-specific recommendations for GWG should be properly addressed.

## Introduction

Prepregnancy body mass index (BMI) and gestational weight gain (GWG) have been known as robust predictors of pregnancy outcomes of the mother and offspring. Excessive GWG is associated with additional adverse health risks, including a greater risk of gestational hypertension, gestational diabetes mellitus, postmature birth, cesarean section, and macrosomia. In contrast, low prepregnancy BMI and inadequate GWG have been linked to small-for-gestational-age infants and premature birth^1–6^. Therefore, appropriate GWG, in addition to proper prepregnancy BMI, has been repeatedly shown to be important to optimize pregnancy outcomes.

Over the past 60 years, the optimal GWG recommendations have been revised several times due to changes in the demographic and nutrition statuses of women in the United States^7–10^. In 2009, the Institute of Medicine (IOM) published the latest revised GWG guidelines based on the World Health Organization (WHO) BMI categories and recommended an optimal GWG of 12.5~18.0 kg for underweight pregnant women (prepregnancy BMI < 18.5 kg/m^2^), 11.5~16.0 kg for normal weight pregnant women (prepregnancy BMI 18.5–24.9 kg/m^2^), 7.0~11.5 kg for overweight pregnant women (prepregnancy BMI 25.0~29.9 kg/m^2^) and 5~9.1 kg for obese pregnant women (prepregnancy BMI ≥ 30 kg/m^2^). The guidelines also recommended the ranges of GWG for the second and third trimesters^10^.

Although the current 2009 IOM GWG guidelines are more rigorous and complete than the previous guidelines, the recommendations were primarily based on Caucasian women; thus, they were more appropriate in the United States and Europe. Therefore, their generalizability has always been controversial worldwide. Moreover, some researchers believe that the 2009 IOM recommendations do not apply to Asian populations. First, maternal anthropometry differs across populations, and the WHO BMI classification criteria are not suitable for Asian populations. Second, individual studies have shown that the GWG recommendations among Asians, such as individuals from Singapore and Japan, vary by national origin, and these divergences suggest that optimal ranges of GWG may be region-specific, population-specific or ethnicity-specific^11^.

Although the 2009 IOM is controversial in terms of its applicability to people other than those from the United States, its GWG recommendations are currently widely used in many countries, such as China, that do not have their own recommendations^11^. In Jan 2018, the Chinese Medical Association published the national guidelines for preconception and prenatal care, but it only duplicated the 2009 IOM recommendations for GWG monitoring^12^. Considering the controversy mentioned above, the generalizability of the guidelines to Chinese women is under dispute. A small number of studies have explored the appropriate range of GWG in Chinese women according to prepregnancy WHO BMI categories, GWG, and various pregnancy outcomes^7,13–15^. However, these studies are concentrated in the Han population, and there is no research on the appropriate GWG for the Tibetan population.

Tibet is located in the plateau area of inland China and has an average elevation of 4,000 meters, a cold climate and thin air. The ethnicity, eating habits and geographical environment of Tibet are very unique in China. Tibetans are the main ethnic group in Tibet, accounting for 94.4% of the total population, and heavy oil, meat and dairy products are characteristics of their diet. Given the variations in race-ethnicity, diet and other factors that may affect GWG, exploring GWG based on the Tibetan population is of great significance for understanding the racial specificity of GWG and providing a reference for the recommended GWG of the Tibetan population. In the present study, we analyzed the relationship between GWG and pregnancy outcomes based on three different BMI classification criteria, including the WHO BMI classification criteria, the WHO Asian BMI classification criteria and the Chinese BMI classification criteria, to propose the appropriate GWG for Tibetans with different BMI conditions and evaluate the appropriateness of the 2009 IOM GWG recommendations for Tibetan women.

## Results

**Figure 1:**
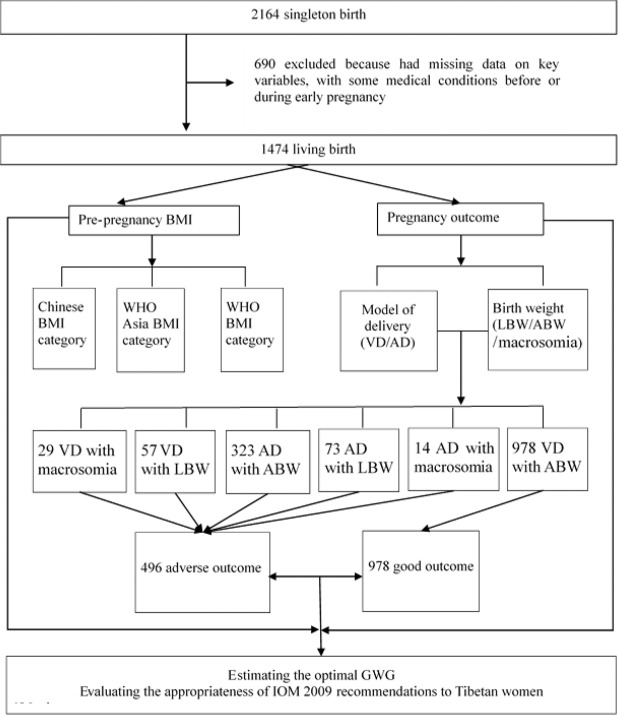
Analytical scheme of present study.

Of the 2164 deliveries in the entire database, 1474 Tibetan singleton deliveries were included in the analysis (Fig. 1). Maternal and neonatal characteristics are summarized in Table 1. Among the study population, most gave birth when they were 20–24 (42.5%) and 25–29 (29.2%) years old. Additionally, 50.5% of the women received an education of 12 years or more. Regarding the prepregnancy BMI of the participants, 244 (16.6%) were underweight, 980 (66.5%) were normal weight, 172 (11.6%) were overweight and 78 (5.3%) were obese based on the Chinese BMI categories; 244 (16.6%) were underweight, 867 (58.8%) were normal weight, 187 (12.7%) were overweight and 176 (11.9%) were obese based on the WHO Asian BMI categories; 244 (16.6%) were underweight, 1054 (71.4%) were normal weight, 159 (10.8%) were overweight and 17 (1.2%) were obese based on the WHO BMI categories. Most women (78.4%) underwent antenatal examination ≥5 times. A total of 1102 (76.6%) women underwent vaginal delivery (VD), and 1301 (88.3%) had appropriate birthweight (ABW) neonates. On average, Tibetan women’s GWG was 12.4 kg. Women aged ≤19 had a higher GWG than women in the other age groups. As BMI increased, GWG showed a decreasing trend, and GWG was positively correlated with neonatal birth weight.

**Table 1:** Distribution of pre-pregnancy BMI, GWG in participants.

Maternal and children characteristics	Number	GWG	
		Mean (SD)	*p* value
Overall	1474	12.4 (6.7)	—
**Age (years)**			
≤19	21 (1.4)	13.7 (10.3)	0.004*
20–24	216 (14.7)	11.6 (6.8)	
25–29	627 (42.5)	13.1 (6.5)	
30–34	430 (29.2)	11.7 (6.7)	
≥35	180 (12.2)	12.2 (6.8)	
**Years of education**			
≤9	629 (42.7)	12.5 (6.8)	0.099
10~12	101 (6.9)	11.0 (9.3)	
≥12	744 (50.5)	12.4 (6.2)	
**Chinese BMI categories** (**kg/m²)**			
Underweight (BMI < 18.5)	244 (16.6)	15.0 (6.2)	0.001*
Normal weight (BMI 18.5–23.9)	980 (66.5)	12.7 (5.8)	
Overweight (BMI 24.0–27.9)	172 (11.6)	9.7 (7.2)	
Obesity (BMI ≥ 28)	78 (5.3)	5.5 (10.4)	
**WHO Asia BMI categories** (**kg/m²)**			
Underweight (BMI < 18.5)	244 (16.6)	15.0 (6.2)	0.001*
Normal weight (BMI 18.5–22.9)	867 (58.8)	12.9 (5.7)	
Overweight (BMI 23.0–24.9)	187 (12.7)	11.2 (6.7)	
Obesity (BMI ≥ 25)	176 (11.9)	7.3 (8.9)	
**WHO BMI categories** (**kg/m²)**			
Underweight (BMI ≤ 18.5)	244 (16.6)	15.0 (6.2)	0.001*
Normal weight (BMI 18.5–24.9)	1054 (71.4)	12.6 (5.9)	
Overweight (BMI 25.0–29.9)	159 (10.8)	8.0 (7.8)	
Obesity (BMI ≥ 30)	17 (1.2)	0.8 (14.4)	
**Antenatal examination**			
0–4 time (s)	319 (21.6)	12.2 (7.3)	0.535
≥5 times	1155 (78.4)	12.4 (6.6)	
**Model of delivery**			
Normal vaginal delivery	1102 (76.6)	12.3 (6.9)	0.354
Assisted delivery	372 (23.4)	12.7 (6.2)	
**Birth weight**			
LBW	130 (8.8)	10.4 (6.0)	0.001*
ABW	1301 (88.3)	12.5 (6.7)	
Macrosomia	43 (2.9)	15.3 (8.9)	
**Outcome**			
Non-adverse outcome	978	12.2 (6.8)	0.300
Adverse outcome	496	12.6 (6.4)	

BMI: body mass index, GWG: gestational weight gain, SD: standard deviation, WHO: world health organization, LBW: low birthweight; ABW: appropriate birth weight. *represents *p* value <0.05, there is statistical difference between groups.

**Figure 2:**
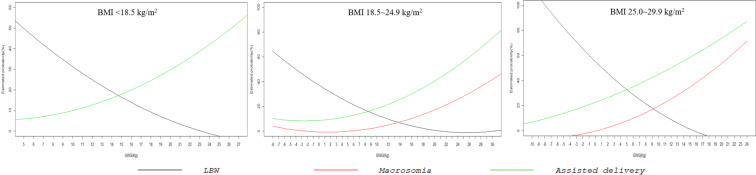
Estimated probability of low birthweight neonates, macrosomia and assisted delivery according to gestational weight gain by WHO BMI categories.

**Figure 3:**
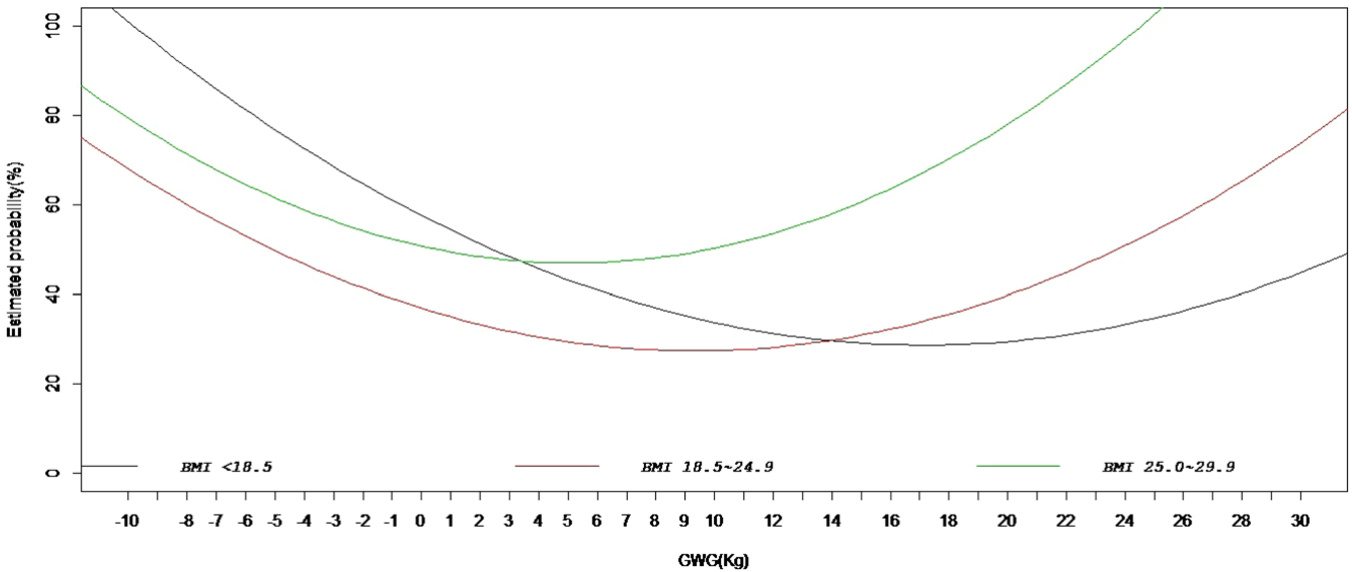
Estimated probability of aggregated adverse pregnancy outcome according to gestational weight gain by WHO BMI categories.

Figure 2 shows the association between GWG and estimated probability of low birthweight (LBW), macrosomia and assisted delivery (AD) in the logistic regression models using GWG as a continuous predictor, stratified by prepregnancy BMI category. In all logistic regression models, the predicted probabilities of LBW decreased as GWG increased, whereas the predicted probabilities of macrosomia and AD increased exponentially with increased GWG, the probabilities grew slower when the GWG value is low, and then increased faster as the GWG value increased. Figure 3 shows the total combined risk of the adverse outcomes (an aggregation of all five adverse categories) according to GWG by prepregnancy WHO BMI category. For all BMI categories, the risk of aggregated adverse pregnancy outcome showed a U-shaped curve with GWG change, which slowly decreased to the lowest point, and then slowly increased. The lowest aggregated risk of adverse outcomes was 28.7% for underweight, 27.4% for normal weight and 46.9% for overweight, and the corresponding GWG values, representing the lowest risk of adverse outcomes, were 17.2, 9.3 and 5.2, respectively.

The optimal GWG range for each WHO BMI category, using the 2009 IOM values for a reference, are given in Table 2. The difference between our proposed Tibetan recommendations and the 2009 IOM recommendations was that for underweight Tibetan women, the optimal GWG rages were considerably higher and wider than those reported in the 2009 IOM guidelines. However, for normal weight and overweight Tibetan women, the optimal GWG was less than those described in the corresponding 2009 IOM categories. Our proposed criteria seem to be more realistic, with more maternal women able to adhere to the recommendation compared to adherence to the 2009 IOM criteria (39.3% vs 38.5% in underweight women; 40.6% vs 34.4% in normal weight women; 28.9 vs 27.0 in overweight women). The estimated optimal GWG values and ranges for each BMI category according to the Chinese BMI classification criteria and the WHO Asian BMI classification criteria are shown in Table 3.

**Table 2:** The differences between IOM and Tibetan recommended weight gain.

WHO BMI categories	IOM 2009	Tibetan
Underweight (<18.5)	12.5~18.0	17.2 (13.3~20.9)
Normal weight (18.5~24.9)	11.5~16.0	9.3 (5.8~12.9)
Overweight (25.0~29.9)	7.0~11.50	5.2 (1.3~9.1)
Obesity (≥30.0)	5.0~9.1	—

IOM: institute of medicine, WHO: world health organization, BMI: body mass index.

**Table 3:** Optimal GWG corresponding to the lowest sum of risk of adverse outcomes.

BMI categories	Chinese BMI categories		WHO Asia BMI categories	
	BMI	Optimal GWG	BMI	Optimal GWG
Underweight	<18.5	17.2 (13.3~20.9)	<18.5	17.2 (13.3~20.9)
Normal weight	18.5~23.9	12.2 (9.4~15.1)	18.5~22.9	13.6 (10.9~16.3)
Overweight	24.0~27.9	6.4 (2.0~10.7)	23.0~24.9	8.4 (4.1~12.7)
Obesity	≥28.0		≥25.0	4.6 (−0.1~9.2)

GWG: gestational weight gain, BMI: body mass index, WHO: world health organization.

In the appendix, the estimated probability of low birthweight neonates, macrosomia and adverse outcomes according to GWG by WHO BMI categories and Chinese BMI categories is shown in Figs. S1 and S2, and the estimated probability of aggregated adverse pregnancy outcomes according to GWG by WHO BMI categories and Chinese BMI categories is shown in Figs. S3 and S4.

## Discussion

Optimal GWG recommendations in the United States have been updated and changed several times in the past 60 years. These changes are mainly based on the following three points: first, the deep understanding of the association between GWG and adverse pregnancy outcomes; second, because of changes in the nutritional status, the prevalence of overweight and obesity has increased among the American population; third, there is also an increase in immigration, and racial specificity needs to be taken into consideration. As we know, the nutritional status and ethnic specificity of Tibetans are different from those in the United States, so assessing the optimal GWG in Tibetans is of great significance in understanding the applicability of the 2009 IOM guidelines to Tibetan women. This is the first study to evaluate the optimal GWG among Tibetan women depend on the probability of meaningful combined outcomes of delivery modes and newborn birth weight, and the determination of the optimal GWG comes from a trade-off of avoiding adverse pregnancy outcomes related to inappropriate GWG.

In the present study, our analyses were consistent with some published work indicating that more GWG was associated with macrosomia and less GWG was with LBW baby^16,17,19^, in addition, the risk of AD increased linearly as GWG increased^17,18^. Our work showed an apparent *J*-curve in risk of AD and macrosomia as compared to weight gain, as GWG increased, the risk increased rapidly, which suggested pregnant women should pay great attention to weight management during pregnancy. In addition, in agreement with the 2009 IOM guidelines, women in lower BMI category could gain more gestational weight than those in higher BMI category. However, it was notable that the optimal GWG in Tibetan women was different from that of the 2009 IOM guidelines. Underweight Tibetan mothers might have more GWG than the 2009 IOM guidelines recommend, and for normal weight and overweight Tibetan mothers, less GWG was recommended. Several reasons may explain the difference. First, our calculating methodology was different with that in other studies, and a quantitative method was used to estimate the optimal GWG in the present analysis. We selected two perinatal outcomes that are closely related to GWG, the combination of neonatal birthweight and delivery modes, as a single composite outcome variable. However, the evaluation of the optimal GWG in the 2009 IOM guidelines was evidence-based, which was based on a range of GWG values correlated to the lowest risk of adverse outcomes of greatest interest, and these outcomes not only related to neonatal weight and mode of delivery, but also included maternal postpartum weight retention, childhood obesity, preterm birth, small or large for gestational age at birth and so on. Second, intrinsic ethnic differences may affect the GWG profile of Tibetan women. Some studies have investigated whether ethnic differences could change the relationship between GWG and perinatal outcomes^17,20,21^, and the conclusions are positive. Consistent with these studies, our findings clearly indicated that it might not be appropriate to continue to use the 2009 IOM recommendations in the Tibetan population, and ethnicity-specific recommendations for GWG should be properly addressed. Third, we speculate that the difference in geographical environment and dietary structure may explain this difference. Tibetan women live in a high-altitude environment where the air is thin, the temperature is low, and people consume large amounts of meat and dairy products. Of course, this is just a speculation and needs further research.

In addition to the WHO BMI classification, our study also assessed the optimal GWG in each BMI group based on the Asian BMI classification. Currently, some researches in the Asian region, Japan, South Korea, Vietnam and Singapore have been conducted to put forth their own GWG recommendations, and in addition to the IOM GWG guidelines, various recommendations have been proposed for different populations^3,17,22,23^. Their GWG recommendations, based on the WHO Asia BMI classification criteria, were different from the recommendations in our present analysis; for instance, Japanese GWG recommendations suggest a less GWG in every BMI group than the GWG recommendations we proposed. We hypothesize that the differences in statistical methods but also minimal ethnic differences could explain the discrepancy, and these divergences may imply that optimal GWG may be population-specific.

The 2009 IOM recommendations suggested that the optimal GWG of obese pregnant women was between 5.0 and 9.1 kg and weight loss was discouraged. However, unlike 2009 IOM recommendations, some studies indicated that for pregnant women who are obesity, weight loss during pregnancy was allowed^11,24,25^. Based on the present analysis, we do not support weight loss during pregnancy; however, our study suggested that for obese women, a less GWG than the IOM guidelines recommended was associated with more favorable pregnancy outcomes. In this study, we could not determine the appropriate GWG for obese Tibetan mothers because of the small sample size in the current study.

### Strengths and limitations

To the best of our knowledge, our study is the first research that proposes an optimal GWG recommendation for pregnant Tibetan women based on prepregnancy BMI categories. This recommendation may help to reduce the incidence of infants with LBW and macrosomia and AD in pregnant Tibetan women. By comparing the discrepancies between the 2009 IOM GWG recommendations and our proposed recommendations, we can initially assess the applicability of the 2009 IOM recommendations in the Tibetan population.

Our study has some limitations. This observational study did not address the long-term effects of GWG, such as weight retention and childhood outcomes. Our recommendations, therefore, intend to reduce only the incidence of LBW, macrosomia and AD, not other short- or long-term unwanted outcomes. Furthermore, because of an insufficient number of obese women in the analyses, the optimal GWG could not be estimated in obese women based on the WHO BMI classification criteria or the Chinese BMI classification criteria. In addition, maternal prepregnancy weight was based on self-reported data, there may be recall bias.

## Conclusion

The present data suggest that it is less than desirable for Tibetan women to adhere to the IOM GWG recommendations to decrease pregnancy complications. Perhaps the GWG recommendations for the Tibetan population may need to take into account racial specificity, as well as geographical environment and dietary differences.

## Methods

### Study population

Our population-based cross-sectional study was conducted in Lhasa General Hospital, a large tertiary general hospital located in the provincial capital of the Tibetan autonomous region, China. Data from 2164 Tibetan inpatients who had singleton births between September 1, 2017, and August 31, 2018 were obtained by trained medical workers. When pregnant women were admitted to the department of obstetrics and gynecology, informed consent was obtained from all subjects, if subjects are under 18, the informed consent was obtained from a parent and/or legal guardian. Administrative permission was provided by the hospital. Ethical approval was given by the medical ethics committee of Lhasa General Hospital. All methods were performed in accordance with the relevant guidelines and regulations.

### Data information

All data used in the present study were from two sources: a maternal case investigation and hospital discharge abstracts. The maternal case investigation was initiated by the National Maternal and Child Health Monitoring Office for hospital monitoring in China. The maternal case questionnaire was used for data collection in the maternal case investigation and mainly included basic information (age, education, occupation, marital status, height, prepregnancy weight, length of hospital stay, hospitalization expenses, discharge diagnosis and past maternal history), present pregnancy-related situation (antenatal examination times, the use of nutritional supplements, premature rupture of membranes, eclampsia, intrauterine distress, gestational hypertension, gestational diabetes mellitus, hemolysis, elevated liver enzymes and low platelet syndrome, intrahepatic cholestasis of pregnancy, amniotic fluid volume, etc.), information about present delivery (maternal weight before delivery, mode of delivery, integrity of placenta and membrane, postpartum hemorrhage, etc.), and information about newborns (sex, birth weight, length, head circumference, birth defects, etc.). Information on weight at delivery, maternal complications and neonatal outcomes was extracted from discharge abstracts. After excluding 690 women who had missing data for height, GWG, delivery methods, or infants’ weight or who had a stillbirth or medical conditions (any chronic respiratory, gastrointestinal, hepatic, renal, hematologic, cardiovascular, metabolic, or mental diseases; pregestational hypertension; or diabetes mellitus) before or during early pregnancy, the analysis sample for this study consisted of 1474 women.

### Maternal anthropometry

Maternal prepregnancy weight was based on self-reported data, height in centimeters was measured on standardized height-measuring stations while subjects stood barefoot, and maternal weight before delivery was measured at the visit nearest to the actual date of delivery on a standardized digital weighing scales by trained medical workers. They were measured to the nearest 0.1 kg or 0.5 cm. Prepregnancy BMI (weight [kg]/height [m]) was calculated using prepregnancy weight and height. BMI was classified using the WHO BMI classification (underweight, <18.5; normal weight, 18.5~24.9; overweight, 25.0~29.9; obese, ≥30.0)^16^, the WHO Asian BMI classification (underweight, <18.5; normal weight, 18.5~22.9; overweight, 23.0~24.9; obese, ≥25.0)^17^ and the Chinese BMI classification (underweight, <18.5; normal weight, 18.5~23.9; overweight, 24.0 to <27.9; obese, ≥28.0)^14^. GWG (kg) was calculated as the difference between the weight before delivery and the prepregnancy weight.

### Pregnancy outcome

The pregnancy outcomes used to determine optimal GWG were birthweight (LBW neonates, ABW neonates and neonates with macrosomia) and model of delivery (AD and VD). Low birthweight neonates, appropriate birthweight neonates and neonates with macrosomia were defined as neonate birthweights <2500 g, 2500~4000 g, and>4000 g, respectively^14^. AD was defined as delivery using forceps, vacuum, or cesarean delivery^18^. Based on combinations of delivery type and neonate birthweight, we created a composite perinatal outcome variable with six mutually exclusive and exhaustive categories^16^: (1) VD with macrosomia; (2) VD with LBW; (3) AD with ABW; (4) AD with LBW; (5) AD with macrosomia; and (6) VD with ABW. A good pregnancy outcome was defined as a term VD of a live infant with ABW; therefore, the last category was the ‘nonadverse’ outcome, and the other five categories were the adverse outcomes.

### Statistical analysis

A polynomial regression model, stratified by prepregnancy BMI category according to the three different BMI classification criteria, was used to assess the association between GWG and the composite perinatal outcome. The “VD with ABW” category, comprising both of the ‘non-adverse’ outcomes, was used as the ‘normal’ reference category in the model^16^. The probability of occurrence of the five adverse outcomes was estimated as a function of GWG within each BMI stratum, then a total risk curve was obtained for each BMI category by summing the probabilities of the five adverse outcome categories^16^. The optimal GWG for a given BMI category was defined as the GWG corresponding to the lowest aggregated risk, the GWG values for which the aggregated risk did not exceed a 5% increase from the lowest aggregated risk were defined as the margins of the optimal GWG range in each BMI category^16^. Statistical analyses were performed using R 3.5.0 version for windows. All p values are two tailed; p < 0.05 was considered to be statistically significant in the analyses.

### Ethical statement

Ethical approval was given by the medical ethics committee of Lhasa General Hospital.

## Supplementary information

Supplementary information.
