# Supplementary material for: Optimal Gestational Weight Gain for Tibetans Based on Prepregnancy Body Mass Index
Source: Sci Rep. 2020 Jul 1;10:10690. doi: 10.1038/s41598-020-65725-3 (PMC7329878; doi:10.1038/s41598-020-65725-3)

**Optimal Gestational Weight Gain for Tibetans Based on Prepregnancy Body Mass Index**

Dajie Chen; Xianxian Zhou; Shijiao Yan; Wenzhen Li; Xueyi Yang; Chuanzhu Lv; Zuxun Lu


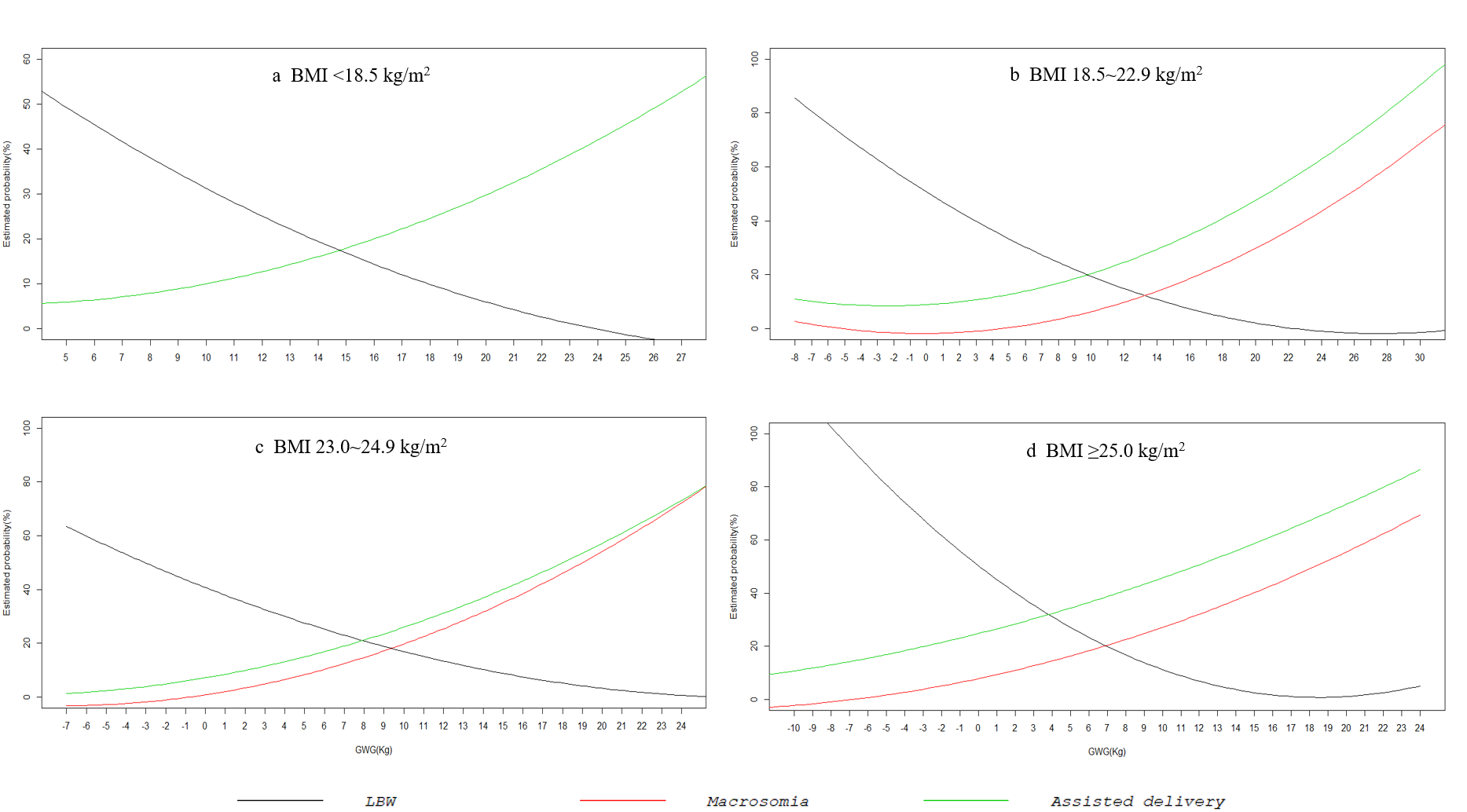


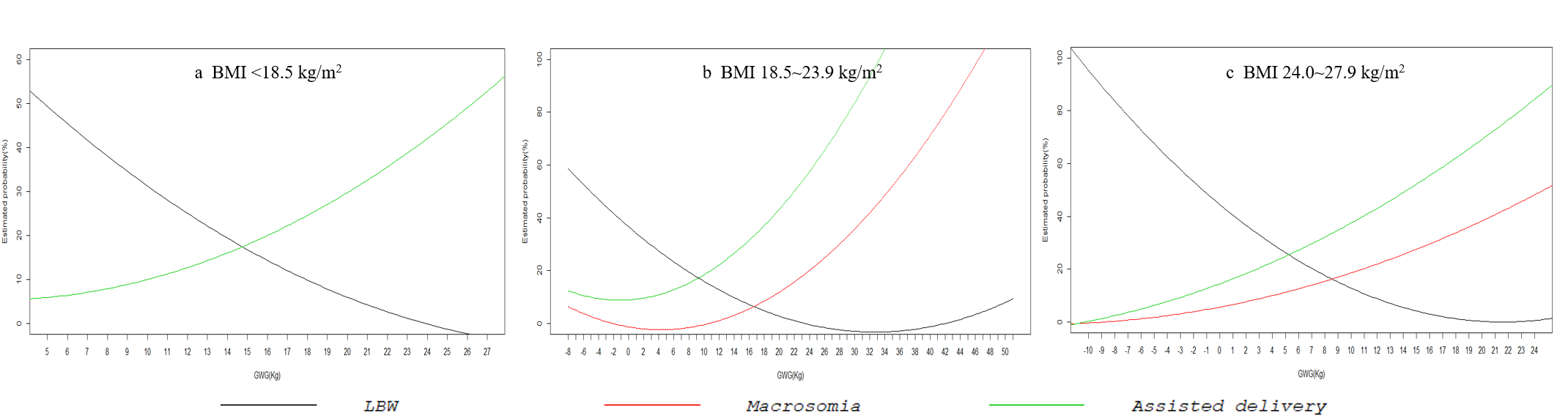


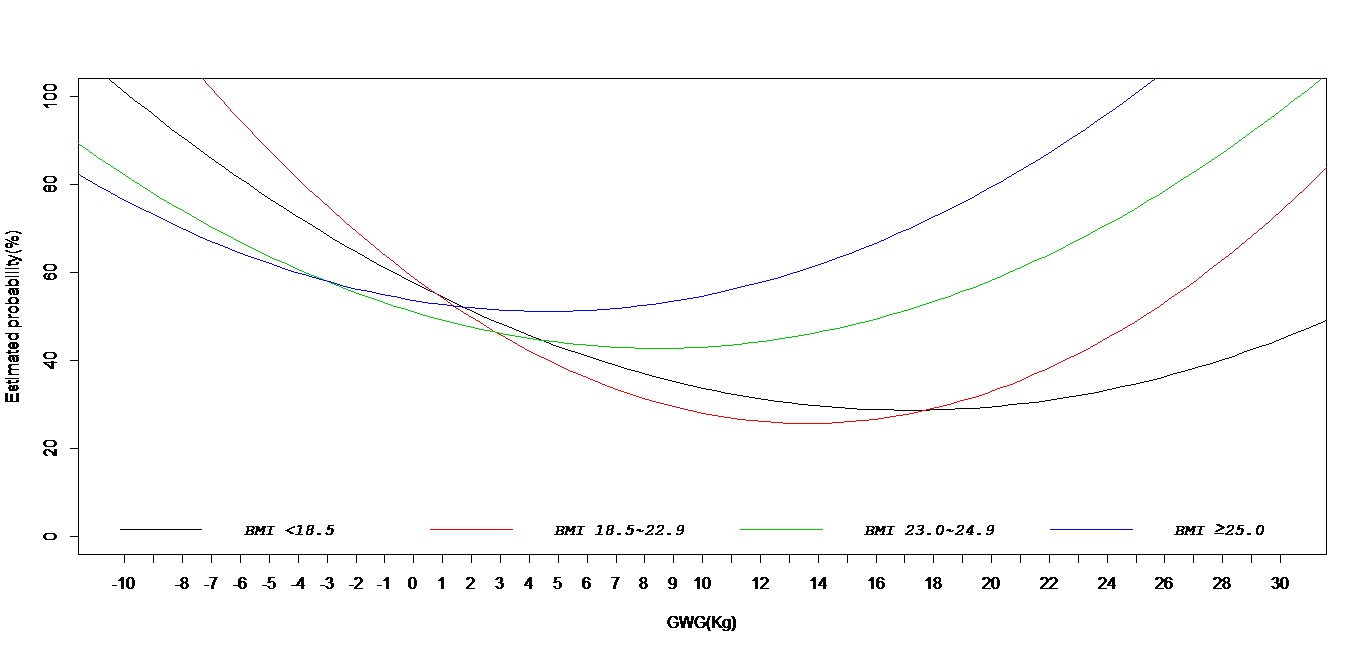


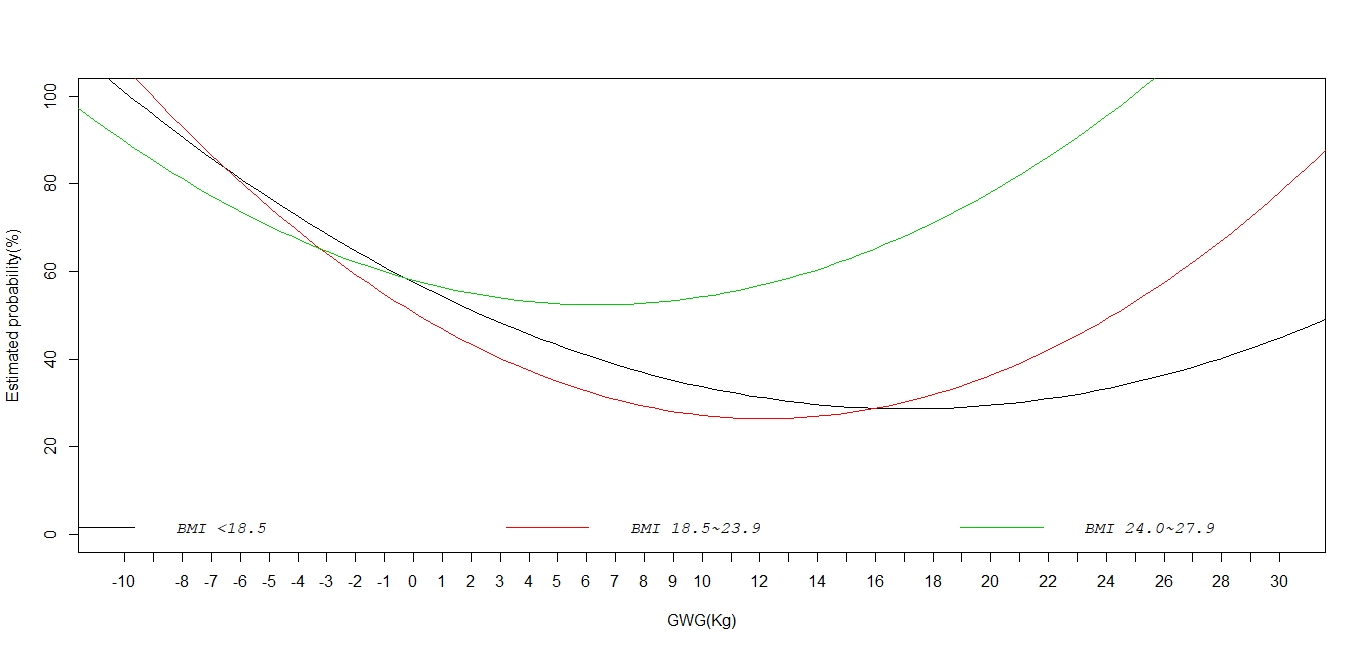

Supplement: Supplementary file 1 — Supplementary information. [file 41598_2020_65725_MOESM1_ESM.docx]
